# Detection of fractures of hand and forearm in whole-body CT for suspected polytrauma in intubated patients

**DOI:** 10.1186/s12891-020-3068-0

**Published:** 2020-01-22

**Authors:** F. Münn, R. A. Laun, A. Asmus, R. Bülow, S. Bakir, L. Haralambiev, A. Eisenschenk, S. Kim

**Affiliations:** 1 Klinik und Poliklinik für Unfall-, Wiederherstellungschirurgie und Rehabilitative Medizin, Universitätsmedizin Greifswald, Sauerbruchstr, 17475 Greifswald, Germany; 2 Abteilung für Hand-, Replantations- und Mikrochirurgie, Unfallkrankenhaus Berlin, Warener Str. 7, 12683 Berlin, Germany; 3 Institut für Diagnostische Radiologie und Neuroradiologie, Universitätsmedizin Greifswald, Sauerbruchstr., 17475 Greifswald, Germany; 4 Leibniz Institut für Plasmaforschung und Technologie (INP Greifswald), Felix-Hausdorff-Str. 2, 17489 Greifswald, Germany

**Keywords:** Polytrauma, Fracture, Sensitivity, Hand, Missed, Delayed, Late, Diagnosis, whole-body CT

## Abstract

**Background:**

The aim of this study was to evaluate the potential of whole-body CT for diagnosis of hand and forearm fractures in intubated patients with suspected polytrauma.

**Methods:**

We performed a retrospective analysis on data collected from two trauma centres in Germany, including demographics, ISS, clinical symptoms, depiction in whole-body CT, and time to diagnosis.

**Results:**

Out of 426 patients included in the study, 66 (15.5%) suffered a hand or forearm fracture. The total number of fractures was 132, the whole-body CT report mentioned 98 (74.2%). 16 (12,1%) fractures of 12 patients were diagnosed later than 24 h after admission. Late diagnoses of fractures of the hand occurred more often if the hand was not fully included in the CT scan field. The sensitivity of whole-body CT for cases with fractures of hand and/or forearm with full inclusion of the corresponding area in the scan field was 80.2%.

**Conclusions:**

This study shows that whole-body CT is a valuable diagnostic tool for hand fractures in polytrauma patients. Hands should be evaluated regardless of clinical presentation in intubated patients after suspected polytrauma if they are included in the whole-body CT.

## Background

In polytrauma, “life before limb” is a premise of treatment [1, 2]. However, the quality of life after trauma and avoidance of long-term ramifications should not be taken lightly [3, 4]. Missing injuries in the examination can put a strain on adequate injury management [5].

Late or missed diagnoses of injuries in polytrauma patients are reported to range between 1.3–65% [6–10] with most of them located in the extremities and spine [5–15]. Comparison of different studies proves difficult due to varying use and definitions of ‘missed’, ‘delayed’ and ‘late’ diagnosis, often used interchangeably [6, 10, 16]. Reasons for late diagnoses can be categorised into unavoidable and avoidable factors [5, 8], a short overview is given in Table 1. Missed injuries with clinical impact were found in 6–15% using whole-body CT (WBCT) without including the arms in CT diagnostics [5, 8, 17]. To lower the risk of missing injuries, special attention should be given to unconscious and intubated patients with severe trauma and brain injuries [16].

**Table 1:** Factors contributing to delayed/missed diagnoses in trauma patients

Unavoidable factors	Avoidable factors
Altered state of consciousness	Reduced attention in high ISS
GCS < 9 and intubation	Insufficient clinical examination
Life threatening injury	Insufficient diagnostic procedures
Injury severity	Wrong interpretation of diagnostic imaging
Injuries not visible in radiographs	Overlooked injuries in diagnostic imaging

Incidence of hand injuries in polytrauma patients ranged between 3.5 and 25% in previous studies [18, 19]. While fractures of the hand and forearm are rarely life threatening, late diagnoses and late presentation to a hand surgeon may result in reduced functionality [1, 4]. In recent years, WBCT has been proven to be a useful tool in trauma diagnostics, allowing identification of most injuries [20].

Regarding WBCT diagnostics, arms are often considered a disturbance for the assessment of the body trunk and thus positioned above the head for abdominal CT. If included in the scan field, it is often not for diagnostic purposes but due to time constraints and fear of iatrogenic injuries [21].

### Aim

We aimed to assess how many fractures of hand and forearm were found within and after 24 h and how often WBCT was able to identify the fracture. We examined the association between diagnosis and clinical symptoms and inclusion of the fractured area. We avoid the term “missed” as we did not assess the number of fractures that were not discovered until discharge.

## Methods

The study is based on retrospective analysis of patient data in two trauma centres in Germany: The first (C1) is a university hospital in Greifswald, a city with a rural catchment area and a population of 57,985 in 2016. The second (C2) is a dedicated trauma centre located in Berlin, a city with a population of 3,574,830 in 2016. We searched the picture archiving and communication system (PACS) for patients that received a WBCT and reviewed the clinical information of the corresponding cases. A WBCT would be performed for the same indication at both centres and according to the German guidelines for the treatment of severely injured patients, including patients with impaired consciousness and suspected trauma history [2].

Patients were included in this study if no previous instrumental diagnostics had been performed and if a tracheal tube was visible in the WBCT, eliminating patient dependent clues and focusing on manual and instrumental diagnostics. WBCT cases for follow-up and for non-traumatic reasons were excluded from the study.

In C1 we identified 939 WBCT cases during the survey period from 1 Jan 2013 to 31 Dec 2015 and in C2 we found 1887 between 1 Jan 2014 and 31 Dec 2015, totalling 2826 cases. Of 535 intubated patients, 426 had a history of suspected or observed trauma and were included. Sixty six patients showed a total of 132 fractures of hand and/or forearm.

For every patient, sex, age, and diagnoses were collected. The Injury Severity Score (ISS) was calculated using AIS 2005 (Update 2008).

The radiology report of the WBCT scan as well as all following medical procedures and corresponding reports of the patient until discharge were analysed for injuries of hand or forearm (phalanges, metacarpal and carpal bones, radius and ulna).

In case of injuries of the hand or forearm, the type of injury, diagnostic modality, clinical description, and time to diagnosis were recorded. We reviewed the admission and discharge documents for clinical symptoms like swelling, bruises, wounds or other pathological findings of the hand and forearm.

We analysed whether fractures were identified through WBCT and set two categories for the time to identification by WBCT or X-ray: “early” within 24 h after admission and “late” after 24 h.

In both centres, the WBCT was performed with contrast agent using a 64-slice device. The radiological report was written by the radiologist on duty and reviewed by a senior radiologist usually within 24 h. The time between first and second assessment, and the years of experience of the radiologists were not available.

For this study, the CT scans were reviewed for position of the hands and forearms and their inclusion in the scan field. If a bone was not fully visible in his entirety, the area was marked as incomplete. The border between the hand and forearm was defined at the radiocarpal joint. Arm position was categorised depending on the location of the hand which could be on the trunk, on the thighs distal of the inguinal ligament, next to the patient, and other.

Quantitative values are shown with median (range).

For sensitivity analysis, the WBCT report on admission was considered the index test. The reference was all known diagnoses on discharge of the patient. True positive was the number of cases with reported fractures in the WBCT. False negative was the number of fractures that were detected later than 24 h after admission. Sensitivity was calculated by dividing the number of true positive cases by the sum of true positive and false negative cases. We did not test for false positive cases. Ninety percent confidence intervals (CI) were calculated using Clopper-Pearson Exact.

Variables were tested for normal distribution using the Shapiro-Wilk test. As it showed a normal distribution only for subgroups, independent continuous variables were tested using Mann-Whitney-U test. Statistical tests of categorial variables with at least 5 expected cases for each field were performed with Chi-square, and Fisher’s exact for tables that did not meet the requirement. A *p*-value of ≤0.05 was defined as significant.

## Results

### Study population

Three hundred sixteen male and 110 female cases had a median age of 52 years (6–94, interquartile range 38). As there was no significant difference for age (*n* = 426, *p* = 0.266, Mann-Whitney-U) and prevalence of fractures of hand or forearm (*p* = 1.000, Chi-square), both groups were pooled for further analysis.

The median ISS of intubated patients with suspected trauma without fracture of hand or forearm was 16 (0–75). Sixty six patients (15%) with fracture of hand or forearm had a significantly higher ISS with a score of 18.8 (5–45) (*n* = 426, *p* = 0.019, Mann-Whitney-U).

### Fractures of hand or forearm

**Fig. 1:**
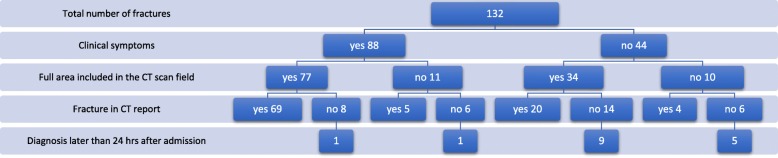
Overview of symptoms, inclusion and diagnosis of fractures

Sixteen of 132 fractures (12%) were diagnosed later than 24 h and belonged to 12 of 66 patients with fracture of hand or forearm (Fig. 1). Late diagnosis was independent of sex (*p* = 0.742, Fisher’s exact) and age (*p* = 0.140, Mann-Whitney-U). The ISS of patients with late and early diagnoses was not significantly different (22 [9–34] vs 17 [5–45], *p* = 0.483, Mann-Whitney-U). Of 16 late diagnosed fractures, 9 required surgical treatment.

### Radiological assessment

Ninety eight of 132 fractures of the hand and/or forearm were documented in the WBCT report resulting in a sensitivity of 74% (CI 66–81%). Fractures with full inclusion in the scan field were reported in 89 of 111 cases (80%). The influence of clinical symptoms and inclusion of the injured area in the WBCT is shown in Table 2.

**Table 2:** Sensitivity of WBCT for detection of fractures of hand and/or forearm

	Clinical symptoms	No clinical symptoms
Area fully included	69/88 fractures 89.6% (80.6–95.4)	20/34 fractures 58.9% (40.7–75.4)
Area not fully included	5/11 fractures 45.5% (16.7–76.6)	4/10 fractures 40.0% (12.2–73.8)

Absolute numbers and sensitivity for different clinical presentation and inclusion in the WBCT scan field. 95%-confidence intervals using Clopper-Pearson exact are given in brackets

Fractures detected later than 24 h after WBCT showed significantly less often clinical symptoms on admission (*n* = 132, *p* <  0.001, Chi-square).

Position of the hand was independent of clinical suspicion (*n* = 132, *p* = 0.139, Chi-square). Fractures of the hand or forearm that were fully included in the scan field were more often diagnosed in the WBCT (*p* <  0.001, *n* = 132, Chi square).

Fractures of the hand were significantly more often diagnosed within 24 h if the hand was fully visible in the WBCT (*p* = 0.002, Fisher’s exact). This was not true for fractures of the forearm and visibility of the forearm in the WBCT (*p* = 1.000, Fisher’s exact). The difference between hand and forearm was significant (*p* = 0.022, Fisher’s exact).

Fractures were not reported in 10% of cases with clinical suspicion and inclusion of the injured area in the scan field.

The hands and forearms were more often fully included when they were placed on the trunk (Table 3).

**Table 3:** Location of the hand and inclusion of hand and forearm of that side in the WBCT scan field

	Area inclusion in WBCT	Hand location			*p*
		Trunk	Thighs	Sides	
Right hand	Complete	67	31	8	< 0.001
	Not complete	5	8	13	
Right forearm	Complete	58	36	9	< 0.001
	Not complete	14	3	12	
Left hand	Complete	64	29	9	< 0.001
	Not complete	10	7	13	
Left forearm	Complete	62	36	11	< 0.001
	Not complete	12	0	11	

The hand and forearms are more often fully included when that respective hand was placed on the trunk. Tests were done using Fisher’s exact test

## Discussion

Diagnosis of fractures of the hand and/or forearm was more often delayed in cases without clinical symptoms and if the affected area was not included in the scan field of the WBCT.

Regarding the factors that contribute to delayed diagnoses (Table 1), we included only intubated patients with a GCS of 3, thus standardising the factors “altered state of consciousness” and “reduced GCS”. Even in reduced GCS, patients might give clues about painful areas which is difficult to categorise.

As delayed diagnoses can be attributed to “life threatening injury” and “injury severity”, as well as “reduced attention in high ISS”, we determined the ISS. While our study sample showed a higher ISS in patients with fractures of the hand and/or forearm compared to those without, the ISS of patients with delayed diagnoses was not higher than that of patients with early diagnoses. This differs to previous studies where hand fractures were associated with reduced injury severity or there was no association found [22, 23].

We searched the admission files for documentation on clinical symptoms, but we could not verify the quality of the examination. In both included centres, symptoms may not have been recorded in writing but still communicated to the radiologist as the trauma team leader will discuss the findings during the WBCT and shortly after. Equally, it is possible that signs of fracture were documented in the admission report but weren’t apparent to the radiologist. Also, we did not examine if injuries were visible in the WBCT. We only differentiated if they were included at all in the scan field.

Full inclusion of the hand and forearm was most often observed when the respective hand was placed on the trunk. Almost 60% of fractures without clinical symptoms were identified if the area was included in the CT scan field. The optimised patient positioning with flexed arms on the chest is not standard in the two analysed centres [24, 25]. Nevertheless, our findings support that this positioning can be recommended for fracture detection.

On the other hand, placement of the hands next to or on the body is not recommended from a radiologist’s point of view because of artefacts and higher radiation dose [21, 24, 25]. Elevation of the arms showed a dose reduction of 3.5 mSv, corresponding to 16–22% [26]. However, this difference was also noted between a 16- and 64- slice CT with a dose reduction of 25% [26].

A study on the clinical impact of arm positioning and image quality showed significantly degraded image quality when arms were placed next to the patient [21]. The authors recommend placement of the arms on the upper abdomen if elevation is not possible. As this study was done on a 16-slice CT, this problem might be addressed by a 64-slice device combined with iterative reconstruction which was shown to reduce beam hardening artefacts [27, 28].

In the diagnostic work-up of trauma patients, time is an important factor to take into consideration. In single-pass WBCTs, arms are not elevated in the sense of a time adapted protocol [29]. In these cases, using the WBCT images for hand and forearm diagnostics creates no further adverse effects. On the contrary it adds gain as we could show, that even without particular attention on hand and forearm diagnosis, 74% of fractures were diagnosed through WBCT. One aspect that still has to be assessed is the visibility of fractures of hand and/or forearm in the WBCT .

As we analysed a subpopulation of all polytrauma patients, the results cannot be directly compared with available publications that included all traumatised patients [18, 19].

Our findings resulted in an increased awareness for injuries of the hand and/or forearm that might be visible in the initial WBCT. While on duty, the senior author discovered fractures of the forearm in the WBCT of two patients shortly after admission that were not reported during the preparation of this manuscript. Elevation of the arms for WBCT is still being discussed in the participating centres.

## Conclusions

Our study population consisted of 426 intubated patients with suspected polytrauma. Fifteen percent had a fracture of hand and/or forearm. Seventy four percent of those fractures were noted in the whole-body CT report. Twelve percent of all fractures of hand and/or forearm were diagnosed later than 24 h. Fractures of the hand were more often diagnosed within 24 h if they were fully included in the scan field.

If hand or forearm are included in the whole-body CT, they should be evaluated in intubated patients after suspected polytrauma. This is true especially in cases without any clinical symptoms that suggest an injury of hand or forearm. Placing the hands on the trunk might be a compromise regarding image quality, radiation dose, time concerns and inclusion of the hands in the scan field.

## Data Availability

The datasets used and/or analysed during the current study are available from the corresponding author on reasonable request.

## Abbreviations

ISS: Injury Severity Score
WBCT: Whole-body CT

## References

[1] Ciclamini D (2014). Particularities of hand and wrist complex injuries in polytrauma management. Injury.

[2] Polytrauma / Schwerverletzten-Behandlung. Registration number 012 - 019, AWMF online 01.07.2016 [cited 01.12.2019]; Available from: https://www.awmf.org/leitlinien/detail/ll/012-019.html.

[3] Kaske S (2014). Quality of life two years after severe trauma: a single-Centre evaluation. Injury.

[4] Mark G (1989). The fate of the polytraumatized patient with a "minor injury" of the hand. Handchir Mikrochir Plast Chir.

[5] Buduhan G, McRitchie DI (2000). Missed injuries in patients with multiple trauma. J Trauma.

[6] Robertson R (1996). Missed injuries in a rural area trauma center. Am J Surg.

[7] Janjua KJ, Sugrue M, Deane SA (1998). Prospective evaluation of early missed injuries and the role of tertiary trauma survey. J Trauma.

[8] Houshian S, Larsen MS, Holm C (2002). Missed injuries in a level I trauma center. J Trauma.

[9] Howard J (2006). Reducing missed injuries at a level II trauma center. J Trauma Nurs.

[10] Thomson CB, Greaves I (2008). Missed injury and the tertiary trauma survey. Injury.

[11] Enderson BL, Maull KI (1991). Missed injuries. The trauma surgeon's nemesis. Surg Clin North Am.

[12] Rizoli SB (1994). Injuries missed during initial assessment of blunt trauma patients. Accid Anal Prev.

[13] Vles WJ (2003). Consequences of delayed diagnoses in trauma patients: a prospective study. J Am Coll Surg.

[14] Pehle B (2006). The significance of delayed diagnosis of lesions in multiply traumatised patients. A study of 1,187 shock room patients. Unfallchirurg.

[15] Eurin M (2012). Incidence and predictors of missed injuries in trauma patients in the initial hot report of whole-body CT scan. Injury.

[16] Pfeifer R, Pape H-C (2008). Missed injuries in trauma patients: a literature review. Patient Safety Surg.

[17] Geyer LL (2013). Incidence of delayed and missed diagnoses in whole-body multidetector CT in patients with multiple injuries after trauma. Acta Radiol.

[18] Ferree S (2017). Fractures and dislocations of the hand in polytrauma patients: incidence, injury pattern and functional outcome. Injury.

[19] Schadel-Hopfner M, Siebert H (2005). Operative strategies for hand injuries in multiple trauma. A systematic review of the literature. Unfallchirurg.

[20] Philipp MO (2003). Radiological emergency room management with emphasis on multidetector-row CT. Eur J Radiol.

[21] Kahn J, Grupp U, Maurer M (2014). How does arm positioning of polytraumatized patients in the initial computed tomography (CT) affect image quality and diagnostic accuracy?. Eur J Radiol.

[22] Schaller P, Geldmacher J (1994). *Die Handverletzung beim Polytrauma. Eine retrospektive Studie an 782 Fällen.* Deutschsprachige Arbeitsgemeinschaft für Handchirurgie ; Deutschsprachige Arbeitsgemeinschaft für Mikrochirurgie der Peripheren Nerven und Gefäße. Vereinigung der Deutschen Plastischen Chirurgen.

[23] Aldrian S (2005). Hand injury in polytrauma. Wien Med Wochenschr.

[24] Hickethier T (2018). Whole-body computed tomography in trauma patients: optimization of the patient scanning position significantly shortens examination time while maintaining diagnostic image quality. Ther Clin Risk Manag.

[25] Karlo C (2011). Whole-body CT in polytrauma patients: effect of arm positioning on thoracic and abdominal image quality. Emerg Radiol.

[26] Loewenhardt B (2012). Radiation exposure in whole-body computed tomography of multiple trauma patients: bearing devices and patient positioning. Injury.

[27] Yasaka K (2017). Full and hybrid iterative reconstruction to reduce artifacts in abdominal CT for patients scanned without arm elevation. Acta Radiol.

[28] Seo N (2018). Feasibility of radiation dose reduction with iterative reconstruction in abdominopelvic CT for patients with inappropriate arm positioning. PLoS One.

[29] Fanucci E (2007). Whole body 16-row multislice CT in emergency room: effects of different protocols on scanning time, image quality and radiation exposure. Emerg Radiol.

